# LoRaWAN Gateway Placement Model for Dynamic Internet of Things Scenarios

**DOI:** 10.3390/s20154336

**Published:** 2020-08-04

**Authors:** Nagib Matni, Jean Moraes, Helder Oliveira, Denis Rosário, Eduardo Cerqueira

**Affiliations:** Computer Science Faculty, Federal University of Pará, Rua Augusto Corrêa 01, 66075-110 Belém, Brazil; jean.anjos.moraes@itec.ufpa.br (J.M.); heldermay@ufpa.br (H.O.); denis@ufpa.br (D.R.); cerqueira@ufpa.br (E.C.)

**Keywords:** LoRaWAN, gateway placement, scalability, resiliency, IoT

## Abstract

Extended Range Wide Area Network (LoRaWAN) has recently gained a lot of attention from the industrial and research community for dynamic Internet of Things (IoT) applications. IoT devices broadcast messages for neighbor gateways that deliver the message to the application server through an IP network. Hence, it is required to deploy LoRaWAN gateways, i.e., network planning, and optimization, in an environment while considering Operational Expenditure (OPEX) and Capital Expenditure (CAPEX) along with Quality of Service (QoS) requirements. In this article, we introduced a LoRaWAN gateway placement model for dynamic IoT applications called DPLACE. It divides the IoT devices into groups with some degree of similarity between them to allow for the placement of LoRaWAN gateways that can serve these devices in the best possible way. Specifically, DPLACE computes the number of LoRaWAN gateways based on the Gap statistics method. Afterward, DPLACE uses K-Means and Fuzzy C-means algorithms to calculate the LoRaWAN gateway placement. The simulations’ results proved the benefits of DPLACE compared to state-of-the-art LoRaWAN gateway placement models in terms of OPEX, CAPEX, and QoS.

## 1. Introduction

The recent explosion of the Internet of things (IoT) [1] technology has been changing society dramatically through disruptive technologies in new sets of verticals and applications, such as smart cities, healthcare, agriculture, environmental monitoring, logistics, home/building automation, smart grid, critical infrastructure monitoring, amongst others [2,3,4]. As a result, forecasts estimate that by 2020 there will be 20.8 billion connected devices [5], the number of 5G-connected IoT devices will reach 4.1 billion by 2024 [6], and 500 billion IoT devices are expected to be connected to the Internet by 2030 [7]. In addition, IoT applications require IoT devices with a long battery life and an inexpensive radio chipset with high coverage and an even less expensive operating cost per device [8].

In this context, Low Power Wide Area Network (LPWAN) has been developed to take advantage of IoT growth to connect the devices [4,9]. LPWAN technologies are of great interest to both the academy and industry for its promising capabilities in broad area connectivity, operating on both licensed and unlicensed frequency bands with the appropriate data rate, power consumption, and throughput tailored to many IoT applications [10]. Consequently, Long-Range Wide-Area Network (LoRaWAN) technology is considered the most adopted LPWAN technology [11], e.g., LoRaWAN Alliance counts with 500+ associated members, as well as 140+ LoRaWAN deployments and 130+ Network Operators in different countries [12]. More specifically, LoRaWAN promises ubiquitous connectivity for many IoT applications, while keeping network structures and management simple.

The LoRaWAN architecture considers a star-of-star topology, guaranteeing single-hop communication between the IoT device and gateway over several channels, and eliminating the need to build and maintain a complex multi-hop network [13]. That is, IoT devices broadcast messages for neighbor gateways that deliver the message to the application server through an existing IP network [14]. Based on such architecture, LoRaWAN provides connectivity to a massive amount of IoT devices deployed over a vast area by employing a control access mechanism with less complexity at the cost of low throughput [15]. One key characteristic of LoRaWAN resides in its dynamic networking features, such as an IoT device’s ability to continually get in and out of the network and to send data with different rate patterns in accordance with application requirements or events [16].

The LoRaWAN gateway has the primary task of providing connectivity for IoT devices, although the possibility remains of IoT devices without a connection due to inefficient network planning [17]. To cope with this issue, it is possible to introduce more LoRaWAN gateways in the environment in order to improve coverage and network performance [18,19]. Nevertheless, deploying LoRaWAN gateways lead to high Capital Expenditure (CAPEX) and Operational Expenditure (OPEX), including the cost of a LoRaWAN gateway, of its leasing, and maintenance. Hence, network planning, such as the LoRaWAN gateways placement model, is a critical problem that influences Quality of Service (QoS) as well as the CAPEX and OPEX [20]. While previous research [20,21,22,23,24,25] has examined LoRaWAN gateway placement problems, to the best of our knowledge, none have considered the minimum network performance along with cost efficiency [23].

Additionally, it is also important to take into account possible physical gateway failures [26] since a LoRaWAN gateway can be compromised, become unavailable, or can even be destroyed due to natural or man-made disasters, reducing the resilience and QoS of LoRaWAN applications [27]. Natural or man-made disasters could compromise correct gateway behaviors by damaging antennas and other equipment by the disruption of the energy supply and others. A resilient and functional network planning plays an important role in LoRaWAN operation. Hence, it is essential to deploy LoRaWAN gateway to mitigate problems related to dynamic LoRaWAN operations and gateway failures, in order to provide high reliability and resilience with low operational costs.

In this article, we introduced a LoRaWAN gateway placement model for dynamic IoT applications called DPLACE. It is common to associate the dilemma of placing LoRaWAN gateways with clustering, where the number of gateways refers to the number of clusters. Accordingly, we divided IoT devices into groups of devices that share some similarity, to allow for the placement of a LoRaWAN gateway that can serve these devices in the best way possible. Based on this assumption, the DPLACE model is comprised of three phases: (i) Determines the amount of clusters for a specific IoT deployment scenario based on Fuzzy C-Means algorithm; (ii) calculates the LoRaWAN gateway location considering the Gap statistics method; and (iii) computes the final LoRaWAN gateway location, that considers a set of LoRaWAN gateway locations for different IoT scenarios in order to account for its dynamism and increase its reliability, resilience, and coverage, while keeping low CAPEX and OPEX. In addition, we further proposed the CAPEX and OPEX for LoRaWAN gateway placement. Finally, we simulated a dynamic IoT environment, which showed the benefits of the DPLACE model in terms of CAPEX and OPEX while keeping the Packet Delivery Ratio (PDR), and Packet Delay at an acceptable level compared to state-of-the art LoRaWAN gateway placement models.

The results obtained here extend those found in preliminary investigation [24]. While previous work [24] computes the LoRaWAN gateway placement in a dynamic IoT scenario, in this paper, we compute the number and positions of LoRaWAN gateways without being aware of the end-devices’ location for a specified area. In this sense, DPLACE considers a set of LoRaWAN gateway locations for different IoT scenarios to apprehend their dynamism, increasing the reliability, resilience, and coverage, while keeping low CAPEX and OPEX. Hence, DPLACE performs better in a scenario with a different number of IoT devices and rate patterns characteristics over time, which is achieved by considering that IoT devices could enter and leave the networks following an approximate Poisson patter. Additionally, we introduced an extended assessment methodology in a more challenging and realistic urban scenario with path loss that accounts for buildings and gateway failure in order to assess how the gateway placement models perform in such a scenario.

Simulation results show that DPLACE has similar behavior in both scenarios with and without LoRaWAN gateway failure, while PLACE [24] reduced the PDR by 3% in a scenario with LoRaWAN gateway failure. By analyzing the results, we conclude that DPLACE improves the PDR by 8% compared to PLACE for the scenario without any failures, while increasing by 10% in a scenario with one gateway failure. However, DPLACE increases the CAPEX and OPEX by 8% compared to PLACE, since DPLACE considers two additional gateways to improve the resiliency and reliability for a dynamic IoT scenario. Therefore, the contributions of this work can be summarized as follows: (i) A LoRaWAN gateway placement methodology for dynamic IoT scenarios and (ii) an extended evaluation that adds new scenarios, metrics, and evaluated protocols.

We organize this article in five section as follows. Section 2 describes the state-of-the art about LoRaWAN gateway placement and their main disadvantages to operate in a dynamic IoT scenario. Section 3 describes the main operations of DPLACE for LoRaWAN gateway placement over a dynamic IoT scenario. Section 4 explains the simulation methodology and the results achieved. Section 5 presents the concluding observations and future works.

## 2. Related Work

This section presents the most recent works focusing on LPWAN gateway placement and planning. Furthermore, in each work, we discuss their advantages and disadvantages to operate in a dynamic IoT scenario. Tian et al. [20] investigated LPWAN gateways placement for a scenario with interference cancellation. This work developed two greedy algorithms in order to find the optimized gateways location. Out of the two algorithms, one is very accurate but computationally complex, while the other is less complex, however, less accurate. Although, this work does not consider the trade-off between QoS, CAPEX/OPEX, and computational cost, and also only evaluates with a lower number of IoT devices.

Gravalos et al. [21] model an Integer Linear Programming (ILP) with the aim to minimize the total network cost by taking into account an IoT device’s information in order to keep the QoS requirements. Specifically, this work computes the gateway placement and transmission information in order to minimize the CAPEX, while respecting predefined QoS requirements. Simulation results on various network topologies and traffic flow estimate the effectiveness of the introduced ILP formulation. However, they did not use LPWAN to prove their algorithm and also do not consider a dynamic IoT scenario.

Rady et al. [22] considered gateway placement in two distinct classes based on specific information of the IoT devices, namely, network-aware and network-agnostic. This work examined two design proposals in both group: (i) Maximize Received Signal Strength Indication (RSSI) based on gateway location and (ii) provide load balance based on RSSI, which gateway should be associated. Nevertheless, this work considers a fixed the number of gateways, leading the gateways to operate with underperformance.

Ousat and Ghaderi [23] considered the problem of planning LoRaWAN in terms of gateway placement and IoT device configuration, considering a mixed-integer non-linear optimization for modeling the plan and deploy LoRaWAN. The simulations used only small networks due to complexity. They developed an approximate algorithm for planning large-scale LoRaWAN efficiently. However, they do not consider the cost.

Matni et al. [24] proposed a LoRaWAN gateways placement called PLACE. It computes the LoRaWAN gateways location using the Fuzzy C-Means (FCM) clustering technique and Gap Statistics to estimate the amount of cluster in each scenario. The objective of the work is to reduce CAPEX and OPEX while maintaining a high PDR. Nevertheless, PLACE does not take into account the dynamism of an IoT network, as well as it was not evaluated in a scenario with gateway failure. In addition, the gateway positioning will vary depending on the coordinates of the device.

Hossain et al. [25] brought out the calculation method from a scenario assumption, network dimensioning to cost structure calculation, which is one main contribution. In the scenario assumption part, the two dimensions, coverage, and capacity are used to divide the scenarios. Based on the assumed scenarios, dimensioning is also carried out from these two aspects. The network is required to meet all the demands. The segments of the cost structure states the cost of deploying an IoT network, the calculation method is also introduced. By using the technique, knowing the input, they can get the output. If they can get more accurate data, the more precise performance analysis can be delivered.

Based on the analyzed works, we conclude that it is important to design an efficient LoRaWAN gateway placement model for a dynamic IoT scenario. However, these works consider neither the network’s dynamism nor the failure of the gateway or both. In addition, they do not examine CAPEX and OPEX costs while providing QoS requirements for a dynamic IoT scenario. Therefore, to the best of our knowledge, all of these decisive characteristics have been not been provided in a unified LoRaWAN gateway placement. Table 1 summarizes the analyzed LoRaWAN gateway placement models.

## 3. DPLACE Model

In this section, we introduce the LoRaWAN gateway placement model for a dynamic IoT scenario called DPLACE. It comprises of three phases (i.e., pre-processing, processing, and validation) to compute the LoRaWAN gateway location, which is explained later. DPLACE aims at improving the packets reception by finding the optimal position of the gateway from various LoRaWAN gateway placements. In addition, to attest to the effectiveness of the system, we introduced well-known heuristics for gateway placement: CAPEX and OPEX.

### 3.1. System Model

By default, the LoRaWAN scenario has three agents: The network server, the gateway, and the IoT device. In this scenario, we have the junction of several gateway positions generated by a gateway placement algorithm. During each execution of a gateway placement, the IoT devices were randomly positioned in the simulation area to collect data and send it to the network server through a gateway. Those IoT devices could enter and leave the networks following an approximate Poisson pattern [16].

We assumed each LoRaWAN gateway to have a circular coverage area *A* with a radio range $R_{j}$, in which a number of IoT devices were deployed and each device could enter and leave the networks following an approximate Poisson pattern that can be used to model aggregated IoT traffic [16]. Each IoT device has an identification i∈[1,N], and a tuple $ED_{i}=\left(x_{i},y_{i},z_{i}\right)$ to represent its geographical coordinates. Moreover, the tuple $GW_{j}=\left(x_{j},y_{j},z_{j}\right)$ represents the gateway geographical coordinates and the transmission power $tx_{j}$ for each LoRaWAN gateway computed by DPLACE with an identification j∈[1,M]. The distance between a given IoT device $ED_{i}$ position and the gateway $GW_{j}$ is computed based on the Euclidean distance $dist_{ED_{i},GW_{j}}$, where $dist_{ED_{i},GW_{j}}\leq R_{j}$ defines that $ED_{i}$ is covered by $GW_{j}$. Table 2 describes the main symbols used in DPLACE modeling.

**Figure 1:**
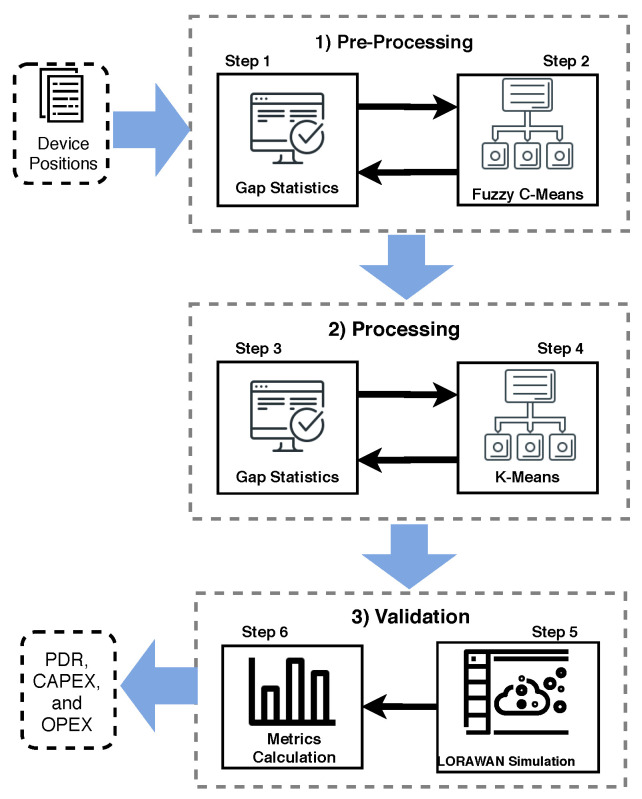
DPLACE overview.

In this approach, the LoRaWAN gateways placement is considered as a cluster assignment problem and by finding the correct number of clusters, we can set the best number of gateways in the simulation. In other words, we partition devices in clusters to minimize outliers, i.e., a device that can not transmit to a gateway. The DPLACE model adopts three phases to compute the LoRaWAN gateway placement, namely, pre-processing, processing, and validation, as shown in Figure 1. In the pre-processing phase, we divide the IoT devices in clusters using the Gap statistic method, resulting in the number of clusters for a specific IoT deployment. Based on the number of clusters, DPLACE applies the Fuzzy C-Means method to compute LoRaWAN gateway placement for the scenario. During the processing phase, we use a set of LoRaWAN gateway locations for different IoT scenarios to apprehend their dynamism, increasing the reliability, resilience, and coverage, while keeping low CAPEX and OPEX. To this end, we cluster the LoRaWAN gateways using the Gap statistic method, then we find the final LoRaWAN gateways location based on the K-Means algorithm. In the validation phase, we attest to the effectiveness of the DPLACE gateway placement previously computed in a LoRaWAN environment.

### 3.2. Pre-Processing Phase

The pre-processing phase is divided into two steps: DPLACE computes the number of gateways based on the the Gap statistics method [28], then it positions the gateways for a specific IoT scenario based on the FCM algorithm. During this phase, we partition devices into clusters so that each device can transmit to a gateway. Hence, it is required to know the number of gateways as input to optimal positioning LoRaWAN gateways.

In light of this, the method to compute the approximated number of gateways consists of a random data set of *N* IoT devices’ geographical coordinates $ED_{i}=\left(x_{i},y_{i},z_{i}\right)$ and the implementation is as follows: Initially, we tested each number of gateways using the gateway index *j* as clusters (j∈[1,2,3…,M]) computing the FCM objective function ($Jm_{j}$) adapted to a LoRaWAN approach, based on Equation (1), which measures how similar the devices are inside the cluster and how different the devices are outside the cluster, i.e., the gateway radius. The variables analyzed are: Gateway index *j*, IoT device identification *i*, membership coefficient $\mu_{ij}$, and the Euclidean distance $dist_{ED_{i},GW_{j}}$ between an IoT device $ED_{i}$ position and a gateway $GW_{j}$.

(1) $${Jm}_{j}=\sum_{j=1}^{M}\sum_{i=1}^{N}{\left(\mu_{ij}\right)}^{m}dist_{ED_{i},GW_{j}}.$$

In addition, we adopted a predefined number of disorganized data sets, i.e., IoT scenarios, where index *b* represents one data set of a total of *B* data sets, where (b∈[1,2,3…,B]), and it is calculated using the FCM objective function ($Jm_{j,b}^{*}$) according to Equation (1) for each data set. Subsequently, the function shown in Equation (2) employs the Gap statistics method to compare the objective function computed for the original IoT scenario ($Jm_{j}$) to the objective function for another random distribution of devices’ positions ($Jm_{c,b}^{*}$), considering the same *N* number of devices. As a result, that step demonstrated how organized our IoT scenario is for each amount of gateways using the GW index *j*, compared to a disorganized scenario. Thus, we use the gateway index *j* to maximize the function value in order to give the average number of clusters:

(2) $$Gap\left(j\right)=\left(1/B\right)\sum_{b}\log\left(Jm_{j,b}^{*}\right)-\log\left(Jm_{j}\right).$$

The Gap statistics (Gap(j)) function usually returns an average value, since it is a possibilistic method. Hence, it is important to consider the standard deviation (sd(j)) for each number of gateways *j*, that is computed based on Equation (3):

(3) $$std\left(j\right)=\sqrt{\frac{\sum_{b}{\left(\log\left(Jm_{j,b}\right)-\frac{1}{B}\sum_{b}\log\left(Jm_{j,b}^{*}\right)\right)}^{2}}{B}}.$$

We evaluate the simulation error to determine the efficiency of the cluster number preference determined by the Gap statistics function (Gap(j)), which is computed based on standard deviation values. Through it, the number of gateways *j* in (Gap(j)), which the gap value with the increase in the number of gateways in our scenario stops growing considerably, i.e., the criterion in Equation (5) is matched, and can perform another approximation to the optimal cluster number. In addition, it is possible to calculate the Simulation Error function ($s_{\left(j\right)}$), which the values that maximize the derived from the Equation (5) the values that best suit this criterion:

(4) $$s_{\left(j\right)}={\mathrm{sd}}_{j}\sqrt{\left(1+1/B\right)}$$

(5) $$Gap\left(j\right)\geq Gap\left(j+1\right)-s_{\left(j+1\right)}.$$

Lastly, the intersection of maximum values of the Gap statistic function Gap(j) and the Simulation Error function $s_{\left(j\right)}$ is calculated according to Equation (5), considering both ends to enhance the accuracy of the selection of the cluster numbers, and consequently determining the least number of gateways using the GW index *j* from this intersection as input to the Fuzzy C-Means algorithm. In Step 2, DPLACE uses FCM to calculate the LoRaWAN gateway location based on the defined number of clusters from the previous step. This heuristic procedure has the benefit of classifying each device $ED_{i}$ with a fractional level of association for each gateway $GW_{j}$, obtaining a device about two or more nearby transmission clusters (overlapping) at different timestamp, such as expected in a LoRAWAN environment that an $ED_{i}$ broadcasts the packet to all the neighboring LoRAWAN gateways. This allows for the improvement of resilience, since as soon as an $ED_{i}$ could not send the message to a gateway, it continues transmitting to others.

The DPLACE algorithm for gateway positioning and for creating a specific network topology is described systematically in Algorithm 1. Initially, random geographic positions for each device $ED_{i}$, the number of gateways *M*, and the ϵ error stop criterion are employed as input. In addition, the membership fraction $\mu_{ij}$ is calculated for each IoT device $ED_{i}$ according to Equation (6), which takes into account the end-device index *i* and GW index *j* to inform whether the device is in the gateway radius. Subsequently, all associations of each device with each gateway is computed in the c-partition matrix $U_{ij}^{r}$ in Equation (8), in which each row represents a device $ED_{i}$ relation with each gateway $GW_{j}$, i.e., the matrix columns. The $U_{ij}^{r}$ matrix is updated using the Euclidean distance $dist_{ED_{i},GW_{j}}$ between the GW and device. In this step, the algorithm analyzes if the device power is enough to transmit based on the sensitivity of each Spreading Factor (SF), defined as $SF=\{sf_{k}|\left(k\in N\right)\wedge \left(7k12\right)\}$, as shown in Table 3 based on [29]. If the signal strength is not enough, the result of the Euclidean distance $dist_{ED_{i},GW_{j}}$ is changed to the maximum distance in the simulation area. The algorithm then repeats the previous steps until it reaches classification convergence as shown in Equation (9), which means that, past a certain iteration, the values in the $U_{ij}^{r}$ matrix nearly stop changing.

Not only is distance used for positioning, but the gateway sensitivity is also considered. After calculating the distance, the RSSI is estimated and compared if the gateways cover all devices considering the LoRaWAN highest signal strength sensibility threshold at Spread Factor 12. In this sense, if many devices are outside the coverage radius, the gateways will change position. With that, the algorithm passes, as a fruit of the pre-processing phase, the definite position of the gateways, which, unitedly with geographic device locations, make up the opening LoRaWAN topology:

(6) $$\mu_{ij}=\frac{1}{\sum_{j=1}^{M}{\left(\frac{∥ED_{i}-GW_{j}∥}{∥ED_{i}-GW_{k}∥}\right)}^{\frac{2}{m-1}}}$$

(7) $$c_{j}=\frac{\sum_{i=1}^{N}{\left(\mu_{ij}\right)}^{m}\cdot ED_{i}}{\sum_{i=1}^{N}{\left(\mu_{ij}\right)}^{m}}$$

(8) $$U_{ij}^{r}=\left[\begin{array}{cccc} 0.002807 & 0.018536 & \ldots & 0.808101\\ 0.002807 & 0.777558 & \ldots & 0.015991\\ \ldots & \ldots & \ldots & \ldots \\ 0.966305 & 0.018536 & \ldots & 0.015991 \end{array}\right]$$

(9) $$∥U_{ij}^{\left(r+1\right)}-U_{ij}^{\left(r\right)}∥\leq \varepsilon \left(\;\mathrm{tolerance}\;\mathrm{level}\;\right)$$

**Algorithm 1:** Fuzzy C-Means **input:** The geographical coordinates of the devices, the desired number of clusters *M* and the error stop criterion ϵ. **output:** The final fuzzy c-partitioned matrix $U_{ij}^{r}$, the geographical coordinates of the gateways, the final Euclidean distance matrix, and the objective function $Jm_{j}$.

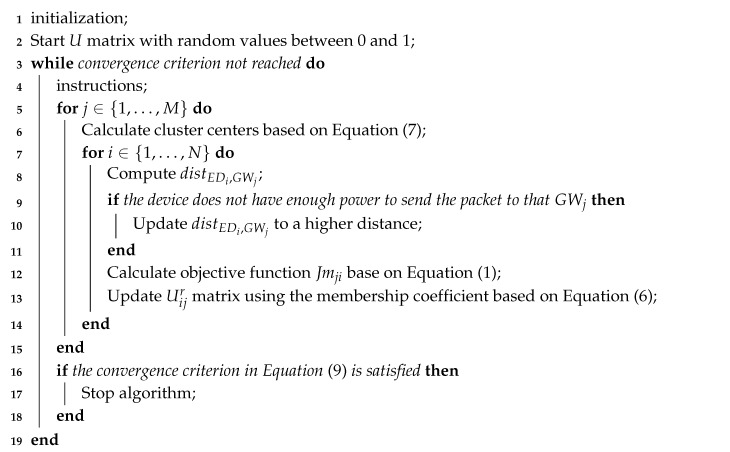



### 3.3. Processing Phase

The processing phase is divided into two steps: The computation of the number of gateways and the gateway placement for a dynamic IoT scenario. In this way, a clustering algorithm has to be executed several times, with different IoT deployments, starting from the pre-processing phase. This creates a set of LoRaWAN gateway placements that are clustered in order to find the optimal LoRaWAN topology.

During this phase, DPLACE uses as input a data set composed of a set of LoRaWAN gateway placements with the gateways’ geographical coordinates $GW_{j}=\left(x_{j},y_{j},z_{j}\right)$. Its implementation comprises of the following steps: It finds out the optimal number of gateways among the data set by making use of the Gap statistics method [28] adapted to the K-Means clustering algorithm. Then, it iterates over each number of gateways using the GW index as the number of clusters (k∈[1,2,3…,K]) and computing the K-Means intra-cluster sums of squares ($J_{k}$), in accordance with Equation (10), which measures the compactness of our clustering. The algorithm processes the GW index *j* of the gateway placement set (considered as a node), the new centroid gateway identification *k*, the Euclidean distance between the node $Gw_{j}$, and the central gateway $C_{k}$:

(10) $$J_{k}=\sum_{k=1}^{K}\sum_{j=1}^{M}{∥Gw_{j}^{\left(k\right)}-C_{k}∥}^{2}.$$

Next, we defined several disorganized data sets of gateways *B* and calculated, for each data set (b∈[1,2,3…,B]), its K-Means intra-cluster sums of squares ($J_{k,b}^{*}$). In light of this, we computed the (Gap(k)) function based on Equation (11) so as to measure how many clusters make our data set organized, where the *k* index, which maximizes the value of this function, gives an estimate of how many gateways should be used:

(11) $$Gap\left(k\right)=\left(1/B\right)\sum_{b}\log\left(J_{k,b}^{*}\right)-\log\left(J_{k}\right).$$

Afterwards, it is necessary to compute the standard deviation (std) based on Equation (3) for each number of gateways (evaluated as a node) to visualize the variation in the Gap(k) values.

Using the standard deviation function (std), we estimated the simulation error Simulation Error function ($s_{\left(k\right)}$) using Equation (4) to validate the precision of the cluster number selection determined by the Gap Statistics (Gap(k)) function. Through it, the number of central gateways *k* in (Gap(k)), which the gap value with the increase in the number of gateways in our scenario stops growing considerably, i.e., the criterion in Equation (5) is matched, can provide another approximation of the number of gateways to use.

Lastly, the intersection of the best values of the Gap Statistic function Gap(k) and the Simulation Error function $s_{\left(k\right)}$, according to Equation (5), are computed to increase the precision of the selection of cluster numbers, which allows the adoption of the least number of gateways, using the GW index *j* from this intersection to serve as input to the K-Means algorithm.

Moreover, DPLACE uses the K-Means algorithm to compute the optimal LoRaWAN gateway placement [22] to manage LoRaWAN dynamism. The K-Means allows for the classification of each node in the data set with a binary degree of membership since it is necessary to better centralize the gateway position within each quadrant of the area. To that end, first DPLACE acquires a set of gateway placements by storing the results of several executions of the pre-processing phase and turns the gateway positions into nodes. Afterward, the algorithm computes the Euclidean distance between a gateway $Gw_{j}$ (defined as a node) and the central gateway $C_{k}$ computed by the K-Means algorithm and returns a small approximation of the optimal value for the number of gateways.

The standard deviation of the value obtained by the Gap function was calculated by Equation (11) and was used to calculate the simulation error function by Equation (12). The obtained value was used to validate the accuracy of the results. The algorithm uses Equation (5) to choose the smallest K value that maximizes the Gap function’s value, consequently producing good results with the fewest possible gateways.

Then, with the algorithm convergence, DPLACE chooses the best position that covers all locations in the simulated area, to ensure that if a new device shows up in the scenario, it is able to transmit to the gateway. The whole process is highly complex and executing the algorithm for each device that enters or leaves the network is not viable. Therefore, when we used K-means to generalize the clusters created during pre-processing, we avoided the need to run for each event in the network. Thus, the result of the processing with the optimal gateway positions and the IoT scenario generated in the pre-processing step is passed to the validation phase.

### 3.4. Validation Phase

The Validation phase is divided into two steps: LoRaWAN simulation and metrics calculation. In step 5, we intended to reinforce the gateway position measured earlier in a LoRaWAN environment. Mainly, it is necessary to include the essential conditions for a LoRaWAN network to operate accurately, such as CR, SF, the number of channels, or even frequency, which are some patterns of the properties of a LoRaWAN. Besides, in this step we include buildings in the scenario, in order to simulate a real urban environment. The LoRaWAN gateway locations require being carried from the last step and evaluated by each algorithm to achieve several packets sent, packets received, number of packets lost due to interference, and the number of dropped packets by not having accessible channels.

In step 6, we wanted to obtain application results and cost of deployment, OPEX, CAPEX, and PDR. CAPEX may be estimated based on Equation (12), which depends on the expenses for acquiring a LoRaWan gateway $C_{Bs}$, the price to deploy the gateway represented by $C_{ins}$, gateway setup (determined by $C_{set}$), and communication installation $T_{xinst}$, which stands for the technology that the gateway will use to communicate with the cloud. The deployment cost $C_{ins}$ refers to the fee required for the local installation of a gateway. Following installation, the new gateway has to be set up, which incurs an additional fee $C_{set}$. These expenses, i.e., $C_{Bs}$, $C_{ins}$, and $C_{set}$, are for a device and interface with a gateway. Nevertheless, there is a cost $T_{xinst}$ to implement gateway-cloud communication. It depends on the local and application, and it could be LTE or fiber:

(12) $$CAPEX=\sum_{i=1}^{S}C_{Bs}+C_{ins}+C_{set}+T_{xinst}.$$

The algorithm has some constraints, for instance, the minimal covered devices’s percentage (value X) in the Equation (13) $A_{k,i}=1$ represents covered devices:

(13) $$\frac{\sum_{i\in I}\sum_{k\in K}A_{k,i}}{\mathrm{TotalUsers}}\geq X.$$

Equation (14) ensures that Shannon’s ability (received by a user from a gateway $Bs_{i}$) must have a minimum value:

(14) $$C_{Bs,i}\geq C_{BSmin}.$$

OPEX is calculated in Equation (15). Particularly, the Operation and Maintenance Cost $C_{man}$, which represents approximately 10% to 15% of the CAPEX cost. Assuming that all the locations in which gateways will be installed were to be leased, $C_{lease}$ stands for the leasing expenses. $C_{elet}$ represents the yearly electricity fee and $C_{Trans}$ refers to the transmission costs, depending on the technology chosen for $T_{xinst}$. All of those are yearly costs, so, to predict future costs, it suffices to alter the variable *t*, that denotes a period in years:

(15) $$OPEX=(C_{man}*CAPEX+\sum_{i=1}^{S}\left(C_{lease}+C_{elet}+C_{Trans}\right))*t.$$

## 4. Evaluation

In this section, we introduce the methodology and metrics used to evaluate the DPLACE model in terms of QoS, CAPEX, and OPEX compared to PLACE and grid deployment of LoRaWAN Gateway.

### 4.1. Methodology

To calculate the amount of LoRaWAN gateways (https://gitlab.com/gercomlacis/cea/lorawan/gap-statistics), we utilize python, from pre-processing to processing phase. We implemented in NS-3 (https://www.nsnam.org/) analyzed gateway placement models with the number and position of the LoRaWAN gateway computed in python. The NS-3 simulator performs the LoRaWAN protocol stack for communication among the LoRaWAN gateway and IoT devices, further applying an error model for LoRaWAN inspired by baseband simulations of a LoRaWAN transceiver over an Additive White Gaussian Noise (AWGN) channel [30]. The implementation of these features permits us to recreate a device performance within LoRaWAN (https://gitlab.com/gercomlacis/cea/lorawan/lorawan-ns3). We conducted 33 simulations with random seeds to the simulator’s pseudo-random number generator (MRG32k3a). Results present the values with a confidence interval of 95%.

In this article, we used a LoRaWAN Class A network with bi-directional communication and considered that the transmissions are started by the LoRaWAN devices, in a non-synchronous way. Class A devices were used because all LoRaWAN devices must implement Class A, whereas Class B and Class C are extensions to the specification of Class A devices. In addition to being the class with the highest energy efficiency. We designate each IoT device a random initial delay after the node creates a new packet every τ seconds. Considering the predominant use of IoT applications admitting only uplink transmissions, i.e., messages from the IoT devices to the gateways, we employed a bi-directional LoRaWAN communication examining only uplink messages to simulate a realistic scenario. Hence, based on the results presented in [31], an increase in the packet received results is expected because of the losses in terms of PDR (Packet Delivery Rate) when we consider downlink messages and re-transmission. In this way, we aim to improve the network data received rate and consequently ensure more excellent reliability.

Three mandatory channels at center frequencies 868.1, 868.3 are required by LoRaWAN. When transmitting, the devices choose one of these three channels randomly. We consider the LoRaWAN gateway radius of 2Km T placed at the height of 30 m, such as expected in an urban scenario. In our simulation, we adopted the European frequency for LoRaWAN, which is 868 MHz utilizing three channels. We consider that all the gateways are communicating with the maximum transmission power P${}_{max}$, and an antenna gain of $G_{t}$. The Gateway failures occur at random times and the failed gateway does not work again. Three different scenarios were simulated to simulate the PDR and the Delay. The first scenario is where all gateways operate throughout the simulation. The second scenario is where only one gateway fails during the simulation, and finally, the last scenario, two gateways, fail during the simulation, and the choice of the gateway to fail was random for each execution. The reason for analyzing more than one GW failure is to verify the algorithms’ behavior in a realistic scenario of a LoRaWAN network deployment with a possible hardware failure of various equipment due to external actions such as climate conditions or electric power drop and oscillation.

For this simulation, we considered 1000 devices uniformly deployed at a height of 1.2 m across a 100 Km${}^{2}$ square area. We considered a smart meter application, where each device must send a message size of 20 Bytes. Each simulation lasted 1200 s and the application period was 600 s. The propagation model chosen was Okumura–Hata, where buildings were implanted to simulate as in an urban environment. The Poisson process approximating had an arrival rate $\lambda =\frac{n}{T}$, where *T* was the sampling period and *n* was the number of asynchronous nodes of the corresponding application. The interarrival time distribution in the Poisson approximated traffic process was exponentially distributed with intensity λ.

We conducted simulations with four different LoRaWAN gateway placement models as follows: Concerning the grid model, we split the scenario within grids of 2.5 Km (designated as Grid16 in the plot), and grids of 2 km (denoted as Grid25 in the plot), and placed each gateway at the center of each cell. PLACE model computes the position of the gateway using only the pre-processing phase introduced in [24]. DPLACE computes the number and positions of LoRaWAN gateways without being aware of the end devices location for a specified area. In this sense, DPLACE considers a set of LoRaWAN gateway locations for different IoT scenarios to apprehend their dynamism, such as introduced in Section 3. In terms of evaluation metric, we estimate the expense in terms of OPEX (Equation (15)), CAPEX (Equation (12)), and total cost for 1 year. We also measure the performance of the LoRaWAN network in terms of PDR.

### 4.2. Results

**Figure 2:**
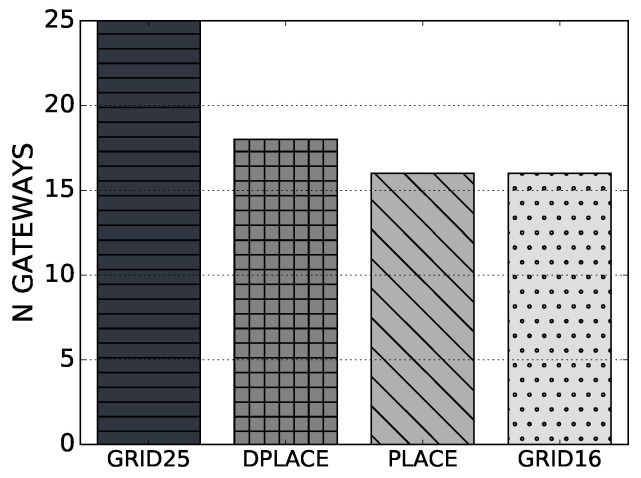
Number of Extended Range Wide Area Network (LoRaWAN) gateways for analyzed LoRaWAN gateway placement models.

The quantity of LoRaWAN gateways for the GRID25, GRID16, PLACE, and DPLACE gateway placement models is shown in Figure 2. By analyzing the results, we conclude that DPLACE gives as a result of 18 LoRaWAN gateways since it calculated the location of gateways and the number of needed gateways based on the device position on different IoT deployment scenarios. In this way, DPLACE aims to give a high PDR, as long as it reduces the CAPEX and OPEX like the following present results. We can also observe that the PLACE model had an average of 15.78 gateways, i.e., ranging from 15 to 16, depending on the IoT deployment scenario. This is because PLACE does not capture the dynamism of the IoT scenario. GRID16 has 16 gateways, which is a similar number of gateways computed by PLACE and DPLACE so as to compare the QoS, CAPEX, and OPEX performance with a similar number of gateways. Finally, GRID25 had 25 gateways and provided a high PDR performance, which can be used to show the cost of the model with such a number of gateways.

**Figure 3:**
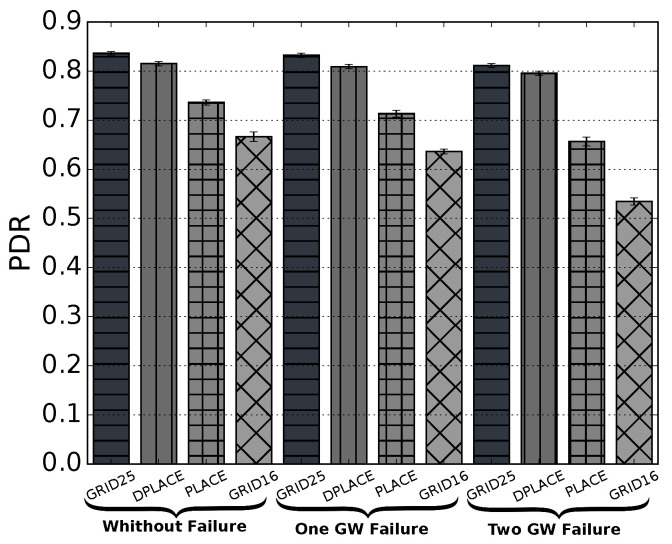
Packet Delivery Ratio (PDR) for analyzed LoRaWAN gateway placement models.

Figure 3 shows the PDR in a dynamic IoT scenario considering grid, PLACE, and DPLACE LoRAWAN gateway placement models in a scenario with one gateway failure, two gateway failure, and without any physical failure. In the failure scenarios, it is observed that an expected higher decrease of packets delivered due to a lack of gateways to cover critical areas. By analyzing the results without LoRAWAN gateway failure, we can observe that 16 gateway placed in a grid have worse PDR compared to the other models, i.e., GRID16 had a 17% lower PDR performance compared with GRID25, and this difference is even more significant in the failure scenarios, with 20% considering one GW failure and 51.8% considering two GW failure. This difference reduces as soon as we compare the other algorithms, GRID16 was 15%, 17%, and 48.1% lower than DPLACE in a scenario with one, two gateways failure, and without failure, respectively. Finally, we compare with PLACE that had the same amount of gateways, the difference is 7%, 13%, and about 8% in a scenario with one, two GW failure, and without failure, respectively.

The PLACE’s PDR is lower by approximately 12% in the scenario with one GW failure, 15% in the scenario with two GW failure, and about 10% in the scenario where all gateways worked properly, in comparison with GRID25. Ultimately, by comparing PLACE to DPLACE, we can see that DPLACE is 8% better for the scenario without any failures, up to 10% if one gateway fails, and up to 13% if two gateways fails. Differently, DPLACE gives related PDR if it compares with the deployment with 25 gateways in a grid, up to 2.5% for the failure of one gateway with seven gateways less, and up to 1.5% for the failure of two gateways. This difference is due to the fact that the GRID25 algorithm needs more gateways to cover the entire area. However, DPLACE computed the optimum number and location of gateway based on the different IoT device location in order to provide high PDR.

By analyzing the results with LoRAWAN gateway failure, we can observe that in a scenario with 100 Km${}^{2}$, only GRID and DPLACE stays close to 80% of PDR. This result is because of two different strategies: The GRID strategy is to place as many gateways as possible since the communications are primary uplink, the interference between gateways are not going to be a problem. However, the project is going to be inviable due to the high cost of implementation. The second strategy is to consider all the possible best positions of the gateways to cluster in a way that suits all possible positions for devices. The result of this clustering is closer gateways. With LoRaWAN technology, the gateway placement compensates for the number of existing buildings. Both the GRID25 and DPLACE PDR were close to 80% due to the scenario applied in the simulation. Throughout the area, there were buildings causing interference in the signal. DPLACE is 10% better than PLACE for the scenario that one gateway fails, 13% better for the scenario where two gateway fails, and only 2% worse than GRID25. It is important to show that a 70% PDR dramatically limits the amount of application that supports this requirement.

One way to understand how devices were distributed is by analyzing the RSSI histogram. Thus it is possible to understand QoS issues, such as delay, better. With this metric, it is impossible to explain packet loss, but only to analyze the signal on the device. The higher the value, the closer to the gateway it is located. At Lora, each SF has a sensitivity limit (Table 3). The higher the SF, the longer the sending time and consequently, the longer the delay. This information was simulated in the scenario of a gateway failure.

**Figure 4:**
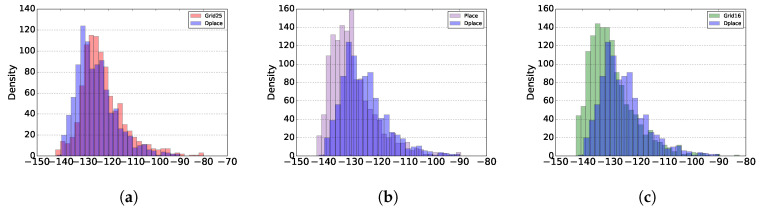
Capital Expenditure (CAPEX) and Operational Expenditure (OPEX) in K for one year. (**a**) Comparison of the Received Signal Strength Indication (RSSI) density of the GRID25 and DPLACE algorithms. (**b**) Comparison of the RSSI density of the PLACE and DPLACE algorithms. (**c**) Comparison of the RSSI density of the GRID16 and DPLACE algorithms.

Figure 4a compare GRID25 with DPLACE, up to −120 dB, the two algorithms are very similar. Between −120 dB and −130 dB, there are many more devices in the Grid25 algorithm compared to DPLACE. The SF9 limit is up to −131. So it can be said that more GRID25 devices were between SF7 and SF9 if compared to DPLACE. Figure 4b shows the comparison between PLACE and DPLACE. Similar to the previous analysis, it is possible to verify a more significant number of devices up to −131 dB of the DPLACE algorithm. In the PLACE algorithm, most devices are from −130 dB. That means that the placement of the gateway causes the devices to be further apart compared to DPLACE. Figure 4c shows the Grid16 algorithm and the distribution works similarly to PLACE. Grid16 has a higher concentration of devices from −130 dB. So DPLACE should have a smaller Time on Air and consequently, a lower Delay.

**Figure 5:**
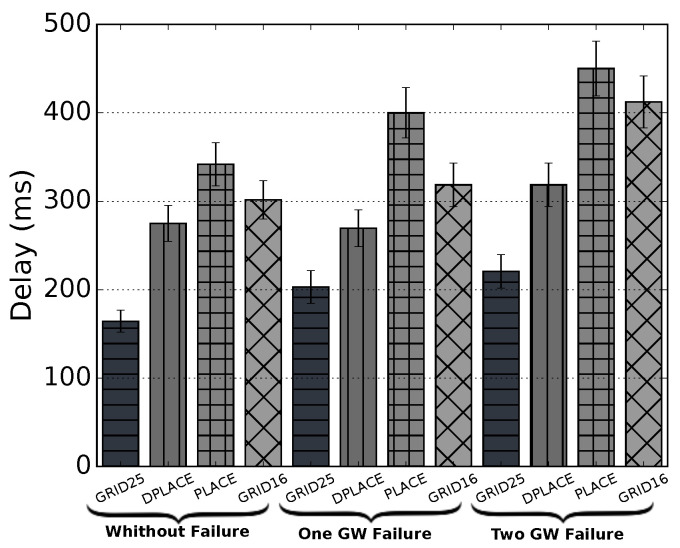
Mean delay for analyzed LoRaWAN gateway placement models.

Figure 5 shows the mean delay using the LoRaWAN time on air (ToA) to compare the models in three different scenarios, scenario with one gateway failure, two gateways failure, and without failure. In the gateway failure scenarios, the devices that are no longer being covered by a gateway are automatically allocated to higher SFs to reach more distant gateways, which results in higher delay. In the scenario with no failure, we can see that GRID25 had a better result, but because of having more antennas, the devices, in general, were closer to the antennas, as shown in Figure 4a. The difference from Grid25 to DPLACE was approximately 40%, while from PLACE to DPLACE, it was 20%, and finally, between Grid16 and DPLACE, it was 12%. For the scenario when a gateway fails, the difference between DPLACE and GRID25 decreased to approximately 24%. The difference between PLACE and DPLACE increased to 33% and GRID16 for DPLACE also increased to 15%. For the last scenario, DPLACE was 30% worse than GRID25, 30% better than PLACE, and 23% better than GRID16.

**Figure 6:**
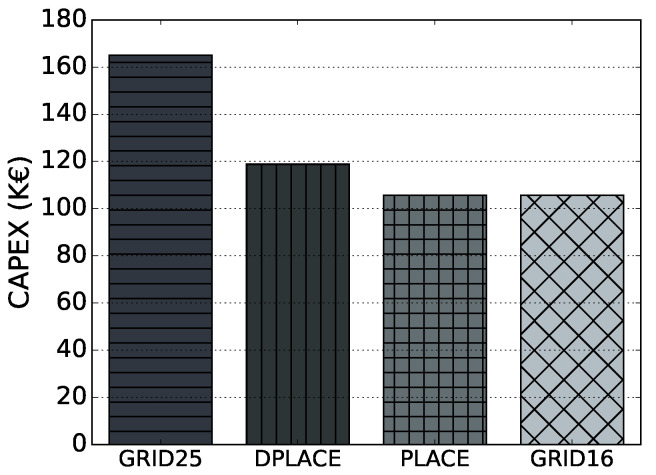
CAPEX for analyzed LoRaWAN gateway placement models.

Figure 6 displays the CAPEX considering GRID25, PLACE, and DPLACE LoRAWAN gateway placement models. We do not consider GRID16 since the PLACE model has an identical amount of gateways and therefore the costs are the same. By investigating the results, DPLACE decreased by 28% the CAPEX, considering it obtained the number of LoRaWAN gateway for a dynamic IoT deployment scenario, while providing similar PDR compared to GRID25. Despite using two more gateways than PLACE, the OPEX of DPLACE was only 8% higher. However, the DPLACE PDR was 10% higher for a failure scenario, so only 8% of OPEX could increase in significant value to the point of being able to cover most applications, considering the dynamics of the network, and reliability.

**Figure 7:**
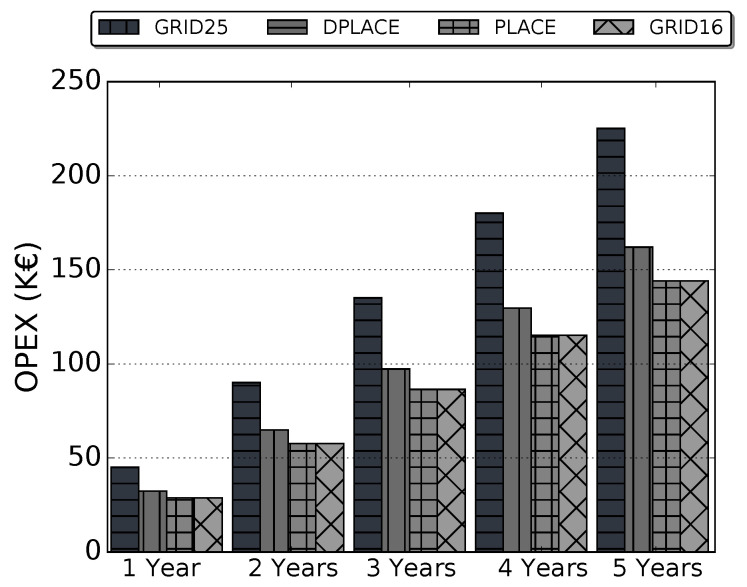
OPEX for analyzed LoRaWAN gateway placement models.

Figure 7 shows the OPEX considering GRID25, PLACE, DPLACE, and GRID16 LoRAWAN gateway placement models. It demonstrates that the difference between DPLACE and GRID25 start in 28% for the first year. We estimate for a time of up to 5 years (t=[1−5]) in Equation (15). Still, even if increase *t*, the difference is equal, as there is no operation to increase or decrease in OPEX calculation. Although DPLACE has an OPEX 8% more expensive than PLACE and GRID16, the DPLACE PDR had a gain of approximately up to 10% compared to PLACE and up to 17% if we compare with GRID16.

**Figure 8:**
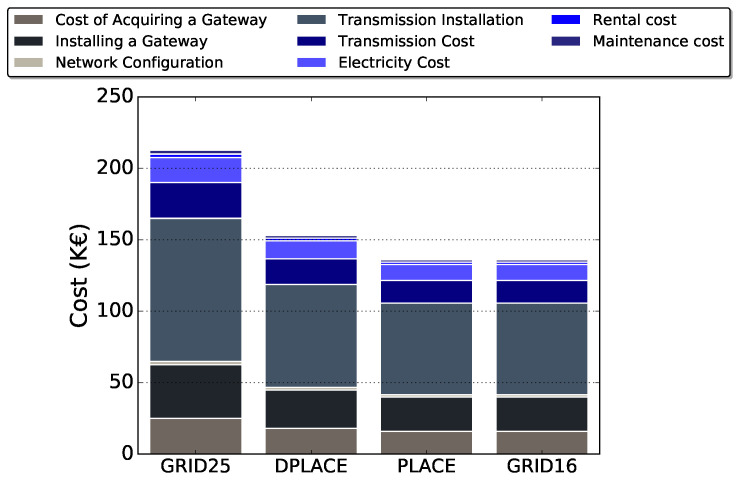
Total cost per specific service for analyzed LoRaWAN gateway placement models.

Figure 8 presents the cumulative cost toward a LoRaWAN gateway placement in a 100 km${}^{2}$ area for a period over one year, that we recognize that DPLACE was 28% more economical than the GRID25. Only the quantity of gateways was being considered because the costs related to the devices will not change despite the chosen algorithm. Since only costs involving gateways are being taken into account. Like CAPEX and OPEX, the rate between DPLACE and PLACE remained the same, 8%.

Figure 8 also shows the costs of both CAPEX and OPEX for each price associated. The cost of installing the transmission was responsible for 47% of the entire cost. This cost is related to the technology that will be responsible for sending the gateway information to a cloud (e.g., LTE, WiFi, etc.). Following the behavior of the previous graphics, PLACE and DPLACE behave in the same way, maintaining even the same relationship between them, PLACE being 8% cheaper. It can be observed that CAPEX costs were generally higher than OPEX costs, which is due to the time used to calculate and in this work it was calculated for one year. If you think about a long term project, it should be used after five years and OPEX costs will be even higher.

## 5. Conclusions

In this article, we proposed the DPLACE model for LoRaWAN gateway placement under a dynamic IoT scenario, which considers buildings, the device’s behavior following the Poisson pattern, and gateway failure for LoRaWAN gateway placement. Originally, the number of gateways was calculated utilizing the Fuzzy C-Means based on a specific IoT deployment scenario; that it was the data for planning the gateway position using Gap statistics. Next, a set of LoRaWAN gateway locations was considered in regards to different IoT scenarios in order to capture the dynamism of such a scenario, increasing the reliability, resiliency, and coverage while keeping a low CAPEX and OPEX. A set of heuristics for LoRaWAN gateway placement was introduced, namely, coverage, QoS, OPEX, and CAPEX. Simulation outcomes attest that the DPLACE model decreased by 28% OPEX and CAPEX compared to the GRID25 and slightly increased the cost in comparison to PLACE. DPLACE also increased the Packet Delivery Ratio (PDR) by approximately 10%, the PDR increased even more when compared to GRID16, reaching 17% for the cost of 8% in the total cost of the project. Therefore, it is worth continuing the investigation in the generalization scope of the spatial algorithm for various technologies and topographies such as industrial sensor networks. In addition, it is valid to implement an algorithm to perform the LoRaWAN configurations on devices, optimizing the network through the gateway positioning and LoRaWAN parameters. Another possible solution is to limit the number of devices per gateway [32] and associate it with our dynamic allocation algorithm. To improve our algorithm, instead of using only the maximum RSSI, we could assess which of the three SF allocation techniques [33] would be best suited for the scenario. 

## Figures and Tables

**Table 1:** Related works summary.

Works	Strategy	Gateway Placement Requirements			
		Dynamism	Reliability	QoS ${}^{1}$	Cost
Tian et al. [20]	Greedy Algorithms	×	✓	✓	×
Gravalos et al. [21]	ILP ${}^{2}$	×	×	✓	✓
Rady et al. [22]	Machine-Learning	✓	×	✓	✓
Ousat and Ghaderi [23]	MINLP ${}^{3}$	×	×	✓	✓
Matni et al. [24]	Machine-Learning	×	×	✓	✓
Hossain et al. [25]	Deployment Framework	×	×	×	✓

${}^{1}$ Quality of Service; ${}^{2}$ Integer Linear Programming; ${}^{3}$ Mixed integer nonlinear programming.

**Table 2:** Main symbols considered for DPLACE modeling.

Symbol	Description
*M*	The maximum number of gateways
*j*	Gateway index (j∈[1,2,3…,M])
$GW_{j}$	Geographical coordinates of a given gateway *j*
*N*	The maximum number of IoT ${}^{1}$ devices
*i*	IoT device index (i∈[1,2,3…,N])
$ED_{i}$	Geographical coordinates of a given IoT device *i*
$Jm_{j}$	Fuzzy C-Means objective function
$\mu_{ij}$	The membership coefficient
*m*	Weighting parameter
$dist_{ED_{i},GW_{j}}$	Euclidean distance between $ED_{i}$ and $GW_{j}$
*B*	Number of disorganized data sets
*b*	A disorganized data set index, where, (b∈[1,2,3…,B])
*r*	The C-partition matrix inde×
$U_{ij}^{r}$	C-partition matrix with all the devices membership
*K*	Number of gateways in Processing phase
*k*	Centroid gateway index in processing phase, where, (k∈[1,2,3…,K])
$C_{k}$	Centroid gateway geographical coordinates computed in processing phase

${}^{1}$ Internet of Things.

**Table 3:** Sensitivity for different Spreading Factor (SF) values and a bandwidth of 125 kHz.

SF	sf${}_{7}$	sf${}_{8}$	sf${}_{9}$	sf${}_{10}$	sf${}_{11}$	sf${}_{12}$
**Sensitivity**	−125	−128	−131	−134	−136	137
